# Deep compressed seismic learning for fast location and moment tensor inferences with natural and induced seismicity

**DOI:** 10.1038/s41598-022-19421-z

**Published:** 2022-09-08

**Authors:** Ismael Vera Rodriguez, Erik B. Myklebust

**Affiliations:** NORSAR, Applied Seismology, Gunnar Randers vei 15, 2027 Kjeller, Norway

**Keywords:** Seismology, Natural hazards

## Abstract

Fast detection and characterization of seismic sources is crucial for decision-making and warning systems that monitor natural and induced seismicity. However, besides the laying out of ever denser monitoring networks of seismic instruments, the incorporation of new sensor technologies such as Distributed Acoustic Sensing (DAS) further challenges our processing capabilities to deliver short turnaround answers from seismic monitoring. In response, this work describes a methodology for the learning of the seismological parameters: location and moment tensor from compressed seismic records. In this method, data dimensionality is reduced by applying a general encoding protocol derived from the principles of compressive sensing. The data in compressed form is then fed directly to a convolutional neural network that outputs fast predictions of the seismic source parameters. Thus, the proposed methodology can not only expedite data transmission from the field to the processing center, but also remove the decompression overhead that would be required for the application of traditional processing methods. An autoencoder is also explored as an equivalent alternative to perform the same job. We observe that the CS-based compression requires only a fraction of the computing power, time, data and expertise required to design and train an autoencoder to perform the same task. Implementation of the CS-method with a continuous flow of data together with generalization of the principles to other applications such as classification are also discussed.

## Introduction

Interest in continuous passive seismic monitoring spans scales from local to global ambits^1^. From industrial applications of fluid injections^2–4^ to regional and global earthquake monitoring^5^, continuous passive seismic monitoring is employed to study the earth’s subsurface and to reduce risks from seismic-related hazards. Early earthquake alerts^6,7^ and tsunami warning systems^8^ rely on a prompt detection and reporting of seismic activity. The same is true for traffic-light systems^9^ developed to control hazards posed by seismicity associated to fluid injections (e.g., hydrofracturing, waste water disposal, CO$_2$ injection). Similarly, among other technologies, the Comprehensive Nuclear-Test-Ban Treaty Organization (CTBTO) will rely on a prompt reporting of seismic events unleashed by nuclear weapon tests to detect treaty violations^10^.

In applications of hazard monitoring, the importance of fast detection and reporting of seismic events is self-evident. But also with the increase of data volumes to analyse, more efficient alternatives to process seismic records are desirable. For instance, consider the surging interest in Distributed Acoustic Sensing (DAS), where fibre optics of several kilometres in length are converted into dense arrays of hundreds if not thousands of seismic sensors^11^. The potential of DAS seismic monitoring has been demonstrated for the study of induced seismicity^12^, natural earthquakes^13,14^ and cryoseismicity^15^. Nevertheless, handling and processing DAS data is computationally demanding as data volumes can quickly reach Terabytes in size. This motivates the development of more efficient data handling and analysis methodologies.

^16,17^Present summaries of methodologies for the analysis of natural and induced microseismicity including the estimation of locations and source mechanisms using full waveforms. Full waveform event location has been approached via imaging methodologies often based on schemes that stack traces transformed via conditioning^17^. Some efficient alternatives are based on stacking along theoretical travel times estimated within a grid of potential locations^18–20^. Other more computationally expensive methods perform reverse time propagation similar to some migration approaches in reflection seismology^21–23^. Source mechanism estimation is frequently detached from event location and performed as a secondary step that requires additional data preparation and uses location as an input^24–26^. On the other hand, full waveform joint location and source mechanism inversion requires the modeling of elastodynamic Green functions^27,28^. Either based on iterative schemes^29,30^ or grid searches^31–34^, these methods have been automated but still face challenges to maintain short response times with dense networks of recording stations.

Strictly speaking, both event location and moment tensor inversion can be achieved with a reduced number of observations if they are of high quality and well distributed around the focal sphere. In practice, sensor deployment may be limited by physical and economical factors, which can hinder the constraining power of the observations. For instance, borehole microseismic monitoring with a single vertical array of receivers cannot constrain full moment tensors and suffers to constrain the azimuthal orientation of the event location^35,36^. Surface microseismic monitoring, on the other hand, has a poor resolution of the vertical coordinate of event locations^37^. Similarly, full moment tensors are not well resolved from surface stations and constraining the isotropic component to zero is common practice to stabilize the inversion^38^. Although generally valid for most natural seismicity, applying this constraint may be limiting for some applications of induced seismicity monitoring, for example, in mining and fluid injections^39,40^. But even if sensors could be freely located around the source (as can be done to some extent in laboratory experiments), identifying a small subset of observations to estimate the source parameters would require inspection of the available records and at least an initial estimation of the event’s location all of which impact turnaround time. As the source location, source mechanism and to some extent the signal-to-noise ratio (SNR) are not known in advance, it is not possible to predict which sensors are best placed to constrain location, and which sample the source radiation pattern in optimal places to constrain the source mechanism. Therefore, it is of advantage to have an automatic system that can simply use all available data to detect events and produce a fast estimation of their source parameters, ideally, with their associated uncertainties.

Like most digital technologies, seismic acquisition and processing tools consider by default signals that are sampled following the Nyquist-Shannon theorem; where a minimum of two samples per period are required to recover the highest frequency component of interest in the signal. However, a new sampling paradigm called Compressive Sensing [CS, see^41,42^] demonstrated that continuous signals can often be sensed using a smaller number of samples than that suggested by the Nyquist-Shannon limit. This entails the measuring of the signals in already compressed form followed by their decompression at a more convenient stage into a Nyquist-sampled version before proceeding with their processing. Benefits of CS technology include hardware simplification and reduced storage requirements as in the single-pixel camera^43^, and reduction of energy consumption and measuring times as in magnetic resonance imaging^44^ and seismic exploration^45^. The main elements required for the application of CS are that the target signal possesses a sparse representation under a dictionary of basis functions, a compression operator with a property called restricted isometry, and a reconstruction (i.e., decompression) algorithm.

Alternative to reconstructing a Nyquist-sampled version of the data, it could be of advantage to infer information directly from the compressed samples^46^. Such an approach, called Compressive Learning, has been investigated in passive seismic monitoring to estimate the location and moment tensor of seismic events^47,48^. But even though Compressed Seismic Learning (CoSeL) successfully detected and estimated seismic source locations and moment tensors, it faced a common drawback in CS applications; this is that the decoding of the information of interest from the compressed signals was time-consuming. In the case of CoSeL, the slow decoding times cancelled out the main potential benefit of the method (i.e., fast response time). Fortunately, advances of recent years in the field of machine learning (ML), and more specifically in deep convolutional neural networks (DCNN), can be used to circumvent this limitation. The resulting protocol and main contribution of this work is referred here as deep Compressed Seismic Learning (deepCoSeL).

ML approaches have been successfully applied in passive seismic for detection, classification and phase-picking of seismic arrivals^49–56^. Other implementations have also targeted estimating event locations, moment tensors and focal mechanisms^57–60^. The objective of the work presented here is to develop a methodology that can be used to detect and/or estimate the source parameters of seismic events. The method must be able to handle large numbers of recording channels with the shortest possible turnaround time, and work in continuous and automatic fashion with little to no user interaction once in processing mode. Depending on the input to the method (i.e., raw waveforms or characteristic functions), its outputs are detections, locations or location and moment tensor. The methodology also places more emphasis on aspects related to data transmission, continuous processing, and network training. Thus, part of the novelties of the proposed deepCoSeL methodology that fulfills our objective are that the incorporation of CS for data compression opens the possibility of more efficient data transmission protocols from the field to the processing center. Also novel in passive seismic processing is a compression protocol that facilitates handling large numbers of seismic records and their processing in the compressed domain, thereby removing the decompression overhead that could impact turnaround time. Additionally, deepCoSeL incorporates a new type of detection function that allows continuous processing instead of relying on pre-identified snapshots of data as most other ML-based methodologies do. Furthermore, the detection function also permits the determination of origin times. User interaction is minimized because deepCoSeL works with a continuous data flow, however, the reliability of the outputs from the model crucially depends on an adequate training and set up.

Another novel aspect worth noting is that deepCoSeL brings together two leading edge technologies into a mutually enabling framework. While the incorporation of a DCNN permits deepCoSeL to fulfill its goal of fast processing, the implementation of CS to compress the training sets that input the DCNN relaxes the computational burden during the training process; thus, facilitating the use of larger training sets that expand larger solution spaces. The latter is made evident in this work by comparing deepCoSeL with an alternative approach using an autoencoder for compression. In the following, a description of the proposed methodology is provided together with a proof-of-concept application with real data from a laboratory experiment where induced seismicity related to fluid injection is investigated.

## Compressed seismic learning (CoSeL)

A condition established in CS theory for its application is that the signal of interest, e.g., ${\textbf{y}}$, can be represented via a linear combination of a sparse number of basis functions from an overcomplete dictionary, ${\textbf{A}}$, this is,

1 $$\begin{aligned} {\textbf{y}} = \textbf{A x}, \quad ||{\textbf{x}}||_0<< N, \end{aligned}$$

where the vector ${\textbf{x}} \in {\mathbb {R}}^{N \times 1}$ specifies which basis functions participate in the representation of ${\textbf{y}}$. Following this requirement, the first step in incorporating CS into the location and moment tensor inversion problem is to develop an adequate sparse parameterization. By using a spatial grid with $N_l$ nodes (or virtual sources) and under the condition that the arrivals of only one source (or a very sparse number of sources compared to $N_l$) are contained in ${\textbf{u}}$,^30^ expressed the source monitoring problem as a block-sparse representation via a linear system of the form

2 $$\begin{aligned} {\textbf{u}} = \textbf{G m}, \end{aligned}$$

where ${\textbf{u}}\in {\mathbb {R}}^{ N_t N_c N_r \times 1}$ is formed with the concatenation of the records of $N_r$ receivers with $N_c$ recording components each with $N_t$ samples; this is, the concatenation of the column-vectors ${\textbf{u}}_{i,j}$, where the subindex *i* runs along the number of receiver-components and the subindex *j* runs along the number of receivers. Matrix ${\textbf{G}}$ is a dictionary of Green functions convolved with a source time function and is formed by $N_l$ six-column blocks, each one linked to a grid node as they are formed by the six Green functions that define all point-source, moment-tensor representations for that particular node or virtual source position [e.g.,^61^]. Under these considerations, solving Eq. (2) produces a block-sparse solution-vector ${\textbf{m}} \in {\mathbb {R}}^{6 N_l \times 1}$, with a support (i.e., six-elements block) that can be directly associated to the source location and with (generally non-zero) values that correspond to the source moment tensor.

**Figure 1:**
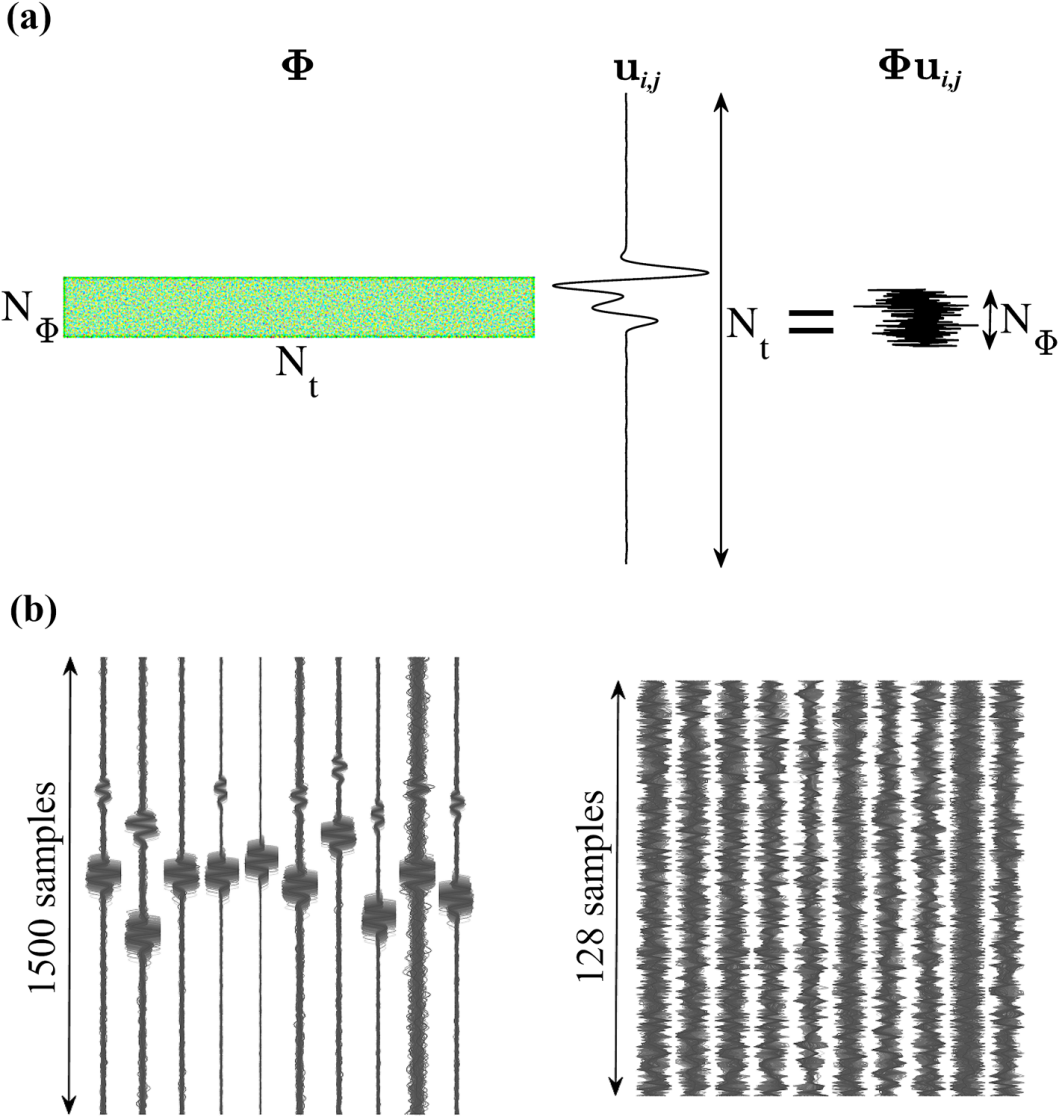
Examples of data compression. (**a**) A seismogram ${\textbf{u}}_{i,j}$ is compressed from ${\mathbb {R}}^{N_t \times 1}$ to ${\mathbb {R}}^{N_\Phi \times 1}$ using an encoding matrix ${\varvec{\Phi }}$ constructed with independent and identically distributed samples drawn from a Gaussian distribution with zero mean and standard deviation of 1/$N_t$. (**b**) Probabilistic pattern of arrivals (left) and a compressed domain representation of it (right) for a source with fixed location and moment tensor. Arrivals were modeled with normal distributions of compressional and shear velocities and contaminated with band-limited Gaussian noise. Variations in SNR are the result of local conditions and the source radiation pattern.

The incorporation of CS into the above parameterization is then accomplished via encoding with a compression matrix ${\varvec{\Phi }}$. For the source monitoring problem the resulting system is called CoSeL and is represented as

3 $$\begin{aligned} {\textbf{u}}_\Phi = {\textbf{G}}_\Phi {\textbf{m}}, \end{aligned}$$

where the subindex $\Phi$ indicates that the time series comprised by the vector or matrix have been encoded with the matrix ${\varvec{\Phi }}$. For the time series of the *i*th component of the *j*th receiver this entails the product ${\varvec{\Phi }}{\textbf{u}}_{i,j}$ (Fig. 1a). Further details about the implementation are found in^48^. The compression matrix acts as a mapping operator moving signals from their original space to a compressed space. The original signal space in time domain is ${\textbf{u}}_{i,j} \in {\mathbb {R}}^{N_t \times 1}$, then it follows that ${\varvec{\Phi }} \in {\mathbb {R}}^{N_\phi \times N_t}$, and the compressed domain is ${\mathbb {R}}^{N_\phi \times 1}$. Clearly, we are interested in $N_\phi<< N_t$.

An important aspect of the encoding process is that the relative *distances* between the compressed signals must be preserved to the extent that they can still be discriminated. This can be accomplished if the compression matrix displays restricted isometry^62^. A straightforward way to construct a compression matrix with restricted isometry is by drawing independent, identically distributed (iid) samples from a Gaussian distribution with zero mean and standard deviation of $1/N_t$^63^.

An advantage of CoSeL over traditional CS implementations is that it solves Eq. (3) to directly extract information from the compressed data; thus, providing an alternative for fast processing while more time-consuming analyses of the uncompressed observations become available. Notice that for the purpose of recovering ${\textbf{u}}$ from the ${\varvec{\Phi }}{\textbf{u}}$ measurements a different dictionary of basis functions would be necessary in place of the Green functions.

Solutions to Eq. (3) are found with sparse solvers [e.g.,^64–66^]. However, an immediate limitation arises from the size of ${\textbf{G}}_\Phi$. Whenever this matrix becomes too big to be held in direct access memory in a computer, the estimation of source parameters suffers a significant degradation in response time. Unfortunately, this is the case in most relevant application scenarios for CoSeL. Fortunately, this bottleneck can be removed with the incorporation of ML into the method.

## Deep learning decoding (deepCoSeL)

The incorporation of ML into CoSeL consists in replacing the sparse solver that provides the source parameter estimations with a DCNN. A similar strategy has recently been investigated in fields ranging from image reconstruction^67,68^ to MRI scanning^69^ and spectroscopy^70^ but never to our knowledge in a seismological application. Thus, the workflow consists of two main steps: data compression followed by moment tensor and event location determination by the DCNN. Additional preprocessing steps may be of advantage depending on specific applications. In the proposed setting, the translation of the computational burden to the training stage of the DCNN allows deepCoSeL to fulfill its goal of fast response time. But the benefits go beyond, with the DCNN providing additional advantages and relaxing other conditions, for example:

1. The problem is changed from a sparse inversion to a pattern recognition, which permits a straightforward generalisation.
2. “Continuous” mapping of the solution space; thus, alleviating inaccuracies in location and moment tensor solutions arising from grid parameterizations.
3. Providing an easy way to account for velocity model inaccuracies, thereby improving robustness in the estimated source parameters.
4. Supplying a practical way to account for noise conditions.

**Figure 2:**
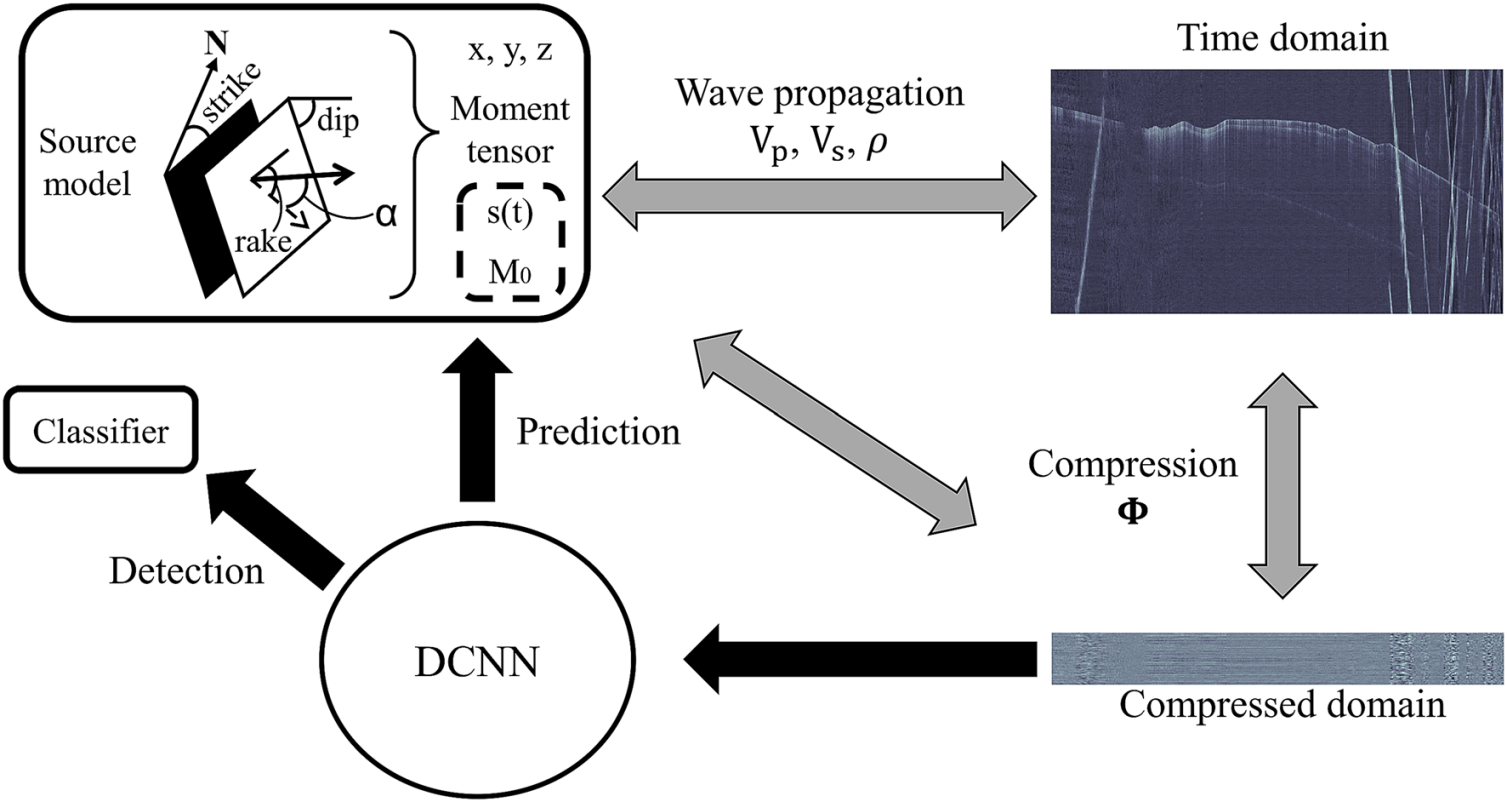
Illustration of deepCoSeL principles. Grey double arrows denote unique correspondences. Since the source model parameters have a unique correspondence with the compressed seismograms, a DCNN can be trained to connect the compressed data to the source model (or a detection classifier). Source parameters within the dashed rectangle have not yet been attempted to recover with deepCoSeL. The number of recoverable source parameters depends on the properties of the time domain traces. For example, removing polarity information before compression would only allow to recover source location.

A seismic source with fixed location and moment tensor produces a pattern of arrivals at a set of recording stations. If the focal coverage is enough, this pattern is unique resulting in an also unique and fully constrained location and moment tensor inversion. In this regard, the most important aspect for deepCoSeL is that the compressed signals retain those unique patterns so that they can be learned by the DCNN (number 1 above). This is accomplished here via encoding with an operator formed with iid Gaussian samples. The DCNN is then trained to connect the (unique) patterns of compressed signals in their input to unique user-defined labels at their output (e.g., location and moment tensor; see Fig. 2). For instance, in the example of application presented here, compressed time domain signals are connected to labels that include the source location and moment tensor. On the other hand, compressed characteristic functions such as short term averages over long term averages (sta/lta) can be connected to the source location. In both cases, the labels could also simply be a classification label in which case the DCNN would only provide detections. In any case, the change of the signals from seismograms to characteristic functions does not require to develop a new, explicit sparse parameterization.

Number 2 in the list is related to an important limitation in CoSeL and every other grid-based method. In the case of CoSeL, since moment tensor inversion relied on the alignment of observations and Green function waveforms, the grid parameterization introduced a trade off between grid resolution for optimal alignment and computational cost. On the other hand, the training sets used for DCNN training are created with a random, uniform sampling of the solution space, and for those regions not sampled, the interpolation capabilities of the DCNN are sufficient to provide a virtually continuous mapping of the location and moment tensor solution spaces.

Numbers 3 and 4 also represent important advantages for deepCoSeL. While in CoSeL Green functions for each grid node are modeled using the best-known, fixed velocity model, training examples for deepCoSeL can be modeled with realizations of velocity models drawn from probability distributions. This strategy generates probabilistic patterns of arrivals (Fig. 1b) that can help with generalization during DCNN training and also to take into account this source of uncertainty in the DCNN estimations. On the other hand, the robustness of the estimations is improved when generating training examples contaminated with varying levels of environmental noise. In contrast, CoSeL and other standard methodologies for moment tensor inversion employ noiseless Green functions.

Applying ML to a seismological problem also brings advantages not often seen in data science. For instance, the existing understanding on source mechanism representations and wave propagation allows to generate ad hoc training sets instead of relying solely on real data examples. This has permitted to investigate how the size of the location and moment tensor solution spaces influences the size of the training sets required to prepare a DCNN with a desired level of prediction accuracy^71^. As the extents of the solution space increase so does the size of the required training set, however, as the compression is also applied to the examples in the training set, the use of CS facilitates the handling of larger sets for DCNN training.

## Detection function for continuous processing

**Figure 3:**
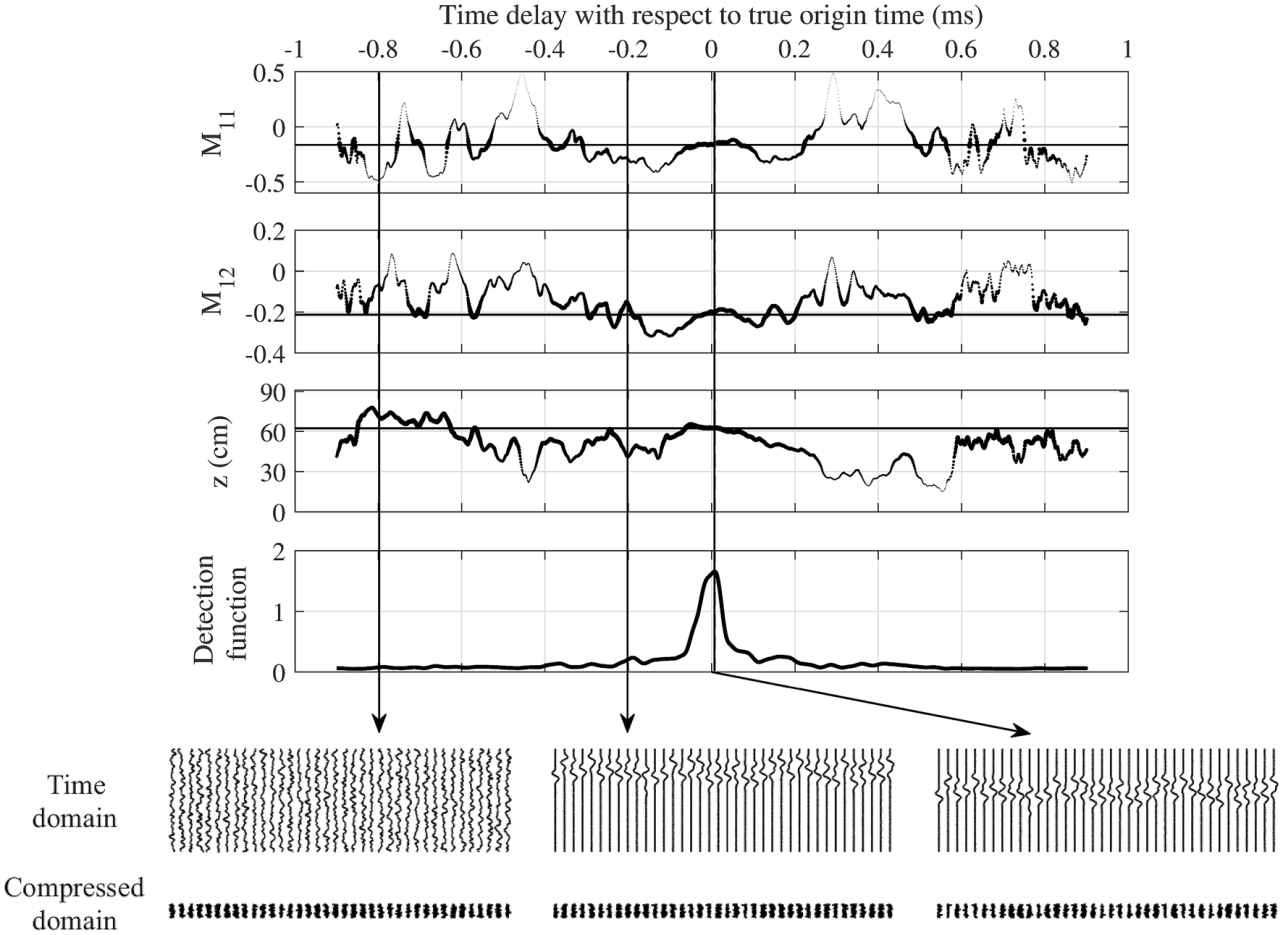
Detection function for deepCoSeL. Top three panels: examples of time series of deepCoSeL predictions (i.e., $p_j^k$) in a continuous processing setting (dotted lines). The size of the dots are increased as the prediction approaches the correct value of the model parameter (horizontal solid lines). Fourth panel from top: detection function constructed with a combination of the nine times series of deepCoSeL predictions. Bottom panels: time domain seismograms (displayed for reference) and their corresponding compressed domain representations (input to DCNN) at three selected positions in time, including at the peak of the detection function. In this example $N_w$ = 30, $N_{ov} = 25$ and $N_{sm} = 40$ samples (see text for details).

Standard detection functions such as sta/lta were developed to detect transients, thus, they are not suited to the properties of the prediction time series that are ouput by the DCNN in deepCoSeL. For this reason, a detection function that exploits the temporal dynamics of the time series of DCNN predictions is described here. If the *j*th sample of the time series of predictions for the *k*th source parameter is $p_j^k$, the *i*th sample of a windowed standard deviation function can be defined as

4 $$\begin{aligned} f_i^k = \sqrt{ \frac{1}{N_w} \mathop {\sum }\limits _{j = N_{ov}(i - 1) + 1}^{j = N_{ov}(i - 1) + N_w} \left( p_j^k - \left[ \frac{1}{N_w} \mathop {\sum }\limits _{j = N_{ov}(i - 1) + 1}^{j = N_{ov}(i - 1) + N_w} p_j^k \right] \right) ^2 }, \end{aligned}$$

where $N_w$ is the length in samples of the processing window and $N_{ov}$ an overlapping. The detection function for the *k*th source parameter is then computed as

5 $$\begin{aligned} \lambda _i^k = \left( f_i^k \cdot df_i^k \right) ^{-1}, \end{aligned}$$

where $df_i^k$ is calculated likewise using Eq. (4) evaluated over the time differential of $p_j^k$. Finally, the detection functions of all the source parameters are combined using a median, and the final result is smoothed with a moving average filter of $N_{sm}$ samples (Fig. 3). Advantages of generating predictions in this way are that there is no need to train the DCNN with only-noise examples and that an approximation for the source origin time is obtained in addition if the training examples are always cut to start from their origin time.

For continuous processing, the system receives chunks of $N_t$ samples which are then compressed trace-by-trace down to $N_\phi$ samples by applying ${\varvec{\Phi }}$. Following that, the compressed traces are input to the trained DCNN and its output predictions fed to an algorithm that generates the median of the curves $\lambda _i^k$ (i.e., the detection function). Finally, the detection function can be triggered by applying a detection threshold.

The input data windows of length $N_t$ can be overlapping. For instance, in the example showed in Fig. 3 the windows have an overlap of $N_t-1$ samples; thus, the detection function in this case displays values at the same sampling rate of the original data. Notice, therefore, that for the purpose of reducing data load for transmission purposes the overlapping must be $< N_t-1$. This introduces a trade off with the capturing of the details of the detection function to be able to identify its peaks. In other words, with less overlap there are less details in the detection function and it becomes harder the task of detecting events.

## Application to seismicity observed during a laboratory experiment

Performance evaluations of deepCoSeL with synthetic data were presented in^71,72^. In particular^71^, investigates a known trade-off in compressed seismic learning. This is related to the compression limit for which the estimation errors are acceptable^47^. The same tradeoff has been observed in simulations with deepCoSeL using compression levels ranging from 0.8% to 12.5% (where 100% is data without compression)^71^. Those results used synthetic examples with the same setting of the real data used in the following part of this work. The compression limit with acceptable estimation errors was located around the 3% compression level in that analysis. Here we use that reference to choose the compression level to test deepCoSeL with real data. In general, it is advisable to investigate on a case-by-case basis the compression level that offers an acceptable estimation error via synthetic analyses.

In the following, deepCoSeL is demonstrated for the detection, location and moment tensor estimation of a set of acoustic emissions (AEs) observed in a triaxial laboratory experiment that investigated induced seismicity from fluid injections. We chose this data type to exemplify the use of deepCoSeL because the monitoring geometry ensures focal coverage in all directions from the sources. Wide focal coverage facilitates full constraining of locations and full moment tensors so that errors in the estimated parameters can more directly be associated to the estimation methodology for its assessment. Note nevertheless that similar to most other methodologies for location and moment tensor inversion, deepCoSeL is agnostic to the scale of the problem and the origin of the seismicity. The most important element to be able to use deepCoSeL in any setting is the capability to model Green functions (and noise) that can be used to reproduce the details of the real data. This is the same requirement needed to perform standard waveform fitting moment tensor inversion.

**Figure 4:**
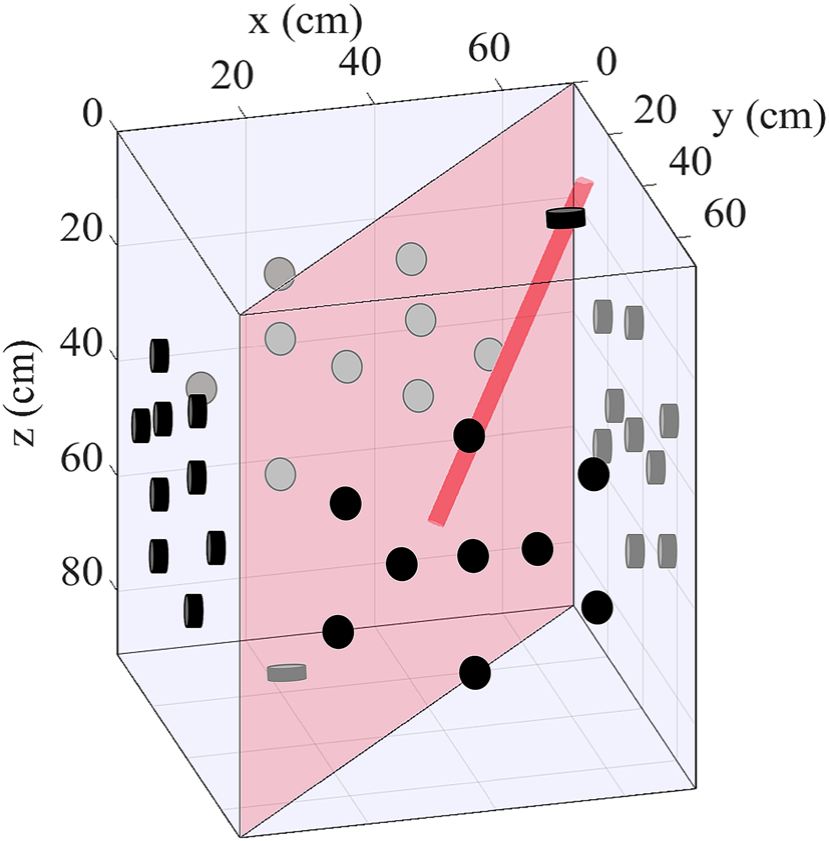
Experimental setting used to illustrate a deepCoSeL application. Black and grey markers represent one-component sensors deployed over the surface of a Castlegate sandstone block. Sensors in grey are situated on the back of the block. The diagonal plane denotes an artificial cut made for the experiment. The cylinder represents a borehole drilled from the top of the block. This borehole was used to inject fluids into the block through an open hole section located near its bottom.

Strictly speaking, the deepCoSeL model is trained to learn full moment tensors without any constraints. However, the training examples are drawn from the general dislocation model^73,74^ with oversampling of dislocations closer to the pure double-couple model. Thus, this is the region of the solution space that we expect the DCNN to learn. The experiment used a Castlegate sandstone block^75^ with dimensions of 71 cm$\times$71 cm$\times$91 cm in the *x*, *y* and *z* directions, respectively. The monitoring network consisted of 38 one-component sensors distributed over the six sides of the block (Fig. 4).

The block had an artificial cut which was ground to remove grooves left by the cutting. After cutting, the two sides of the block were left to dry over three days with hot air blowers. Additionally, a 2.69 cm diameter borehole was drilled at an angle starting from the top face (see Fig. 4). The borehole was cased except for an open hole section of 15.24 cm followed by a 2.54 cm epoxy plug located at the bottom.

The experiment consisted of a total of 22 stages, in which triaxial stress changes were combined with fluid injection cycles from the borehole until slip was induced along the artificial cut^76,77^. Here, a subset of AEs detected during the first stage of the experiment were used to investigate the performance of deepCoSeL. In this first stage, the experimental procedure consisted of increasing triaxial stress homogeneously up to 15.17 MPa, and subsequently injecting 26 liters of a 40 cP fluid to saturate the sample. During saturation, ultrasonic transmission signals were emitted from a subset of the sensors and detected with the remaining instruments. These signals were analyzed to obtain an interpretation of the saturated zone within the block. After finishing with the sample saturation, the stress along the *y* and *z* directions were gradually and homogeneously reduced down to 12.41 MPa. The selected AEs were recorded during this stress relaxation period.

During the experiment, the acquisition system was triggered every time an event was detected. Afterwards, time picks were automatically generated using the Akaike information criterion^78^. Locations were then estimated via an iterative process that minimized travel time residuals using the downhill simplex algorithm^79^. At every iteration, inconsistent time picks with the larger discrepancies were systematically removed to improve the final overall location residuals. The method also takes into account the varying nature of the block velocities in response to the imposed stress variations^80^. This is a state-of-the-art method used in multiple previous projects^81–84^; therefore, we use it as a benchmark to compare the location part of the solution from deepCoSeL.

The source mechanisms of the AEs were investigated in post-processing with a waveform fitting methodology that does not use compression. Waveform fitting moment tensor inversion in this case is challenging due to the resonant nature of the sensors which affect amplitude fidelity. In addition, the heterogeneity and time variation of the velocities in the medium complicate waveform matching. Furthermore, the large number of AEs normally detected in laboratory experiments make unpractical individual analysis. The method employed here was developed attending at these obstacles, it is semiautomatic and makes use of the large number of AEs to derive statistical corrections to the observations^85^. It consists in estimating station corrections for individual P- and S-phases to optimize waveform matching. This is followed by a statistical analysis to create an empirical deconvolution operator that corrects for the instrument response taking into account in-situ effects. The method also incorporates a bias correction for angular sensitivity of the sensors; however, the number of AEs available in this case was insufficient to obtain stable results. With the waveform fitting of individual phases optimized and the instrument response corrected, the method performs least squares full moment tensor inversion without any further constrains or assumptions. The results from this procedure were used to compare with the source mechanism estimations from deepCoSeL.

### Evaluation of the deepCoSeL model

The steps followed for the preparation of the training, validation and testing sets, DCNN architecture and its training are described in the “Methods” section. The compression used was 6.25% (100% is data without compression) and was chosen based on previous analyses with synthetics^71^. This resulted in a training set of 26.9 Gigabytes. In comparison, the Nyquist-sampled training set would be on the order of 430 Gigabytes in size.

The performance of the trained deepCoSeL model incorporating the detection function was assessed using a test set of 2000 synthetic examples with varying levels of SNR. The parameters used to construct the detection function were $N_w = 100$, $N_{ov} = 40$ and $N_{sm} = 100$ and were defined by trial-and-error. Location errors were evaluated with the euclidean distance from the known positions. The source model considered here is that of a general dislocation defined by the angles of strike, dip, rake and $\alpha$^73,74^. The angle $\alpha$ defines the deviation of the displacement vector from the pure double couple case (i.e., $\alpha = 0$; a schematics of a general dislocation model is also displayed in Fig. 2). Dislocation angles errors were evaluated with the formula $eA = 5 \sqrt{ ( \sin \theta _{true} - \sin \theta _{pred})^2 + ( \cos \theta _{true} - \cos \theta _{pred})^2}$, where $\theta _{true}$ and $\theta _{pred}$ are a true and deepCoSeL predicted dislocation angle, respectively. The error computed in this way removes ambiguities in strike and rake angles, and is bounded to the range from zero to ten. For one example, the dislocation angles error is the mean of the errors for the four angles computed this way.

**Figure 5:**
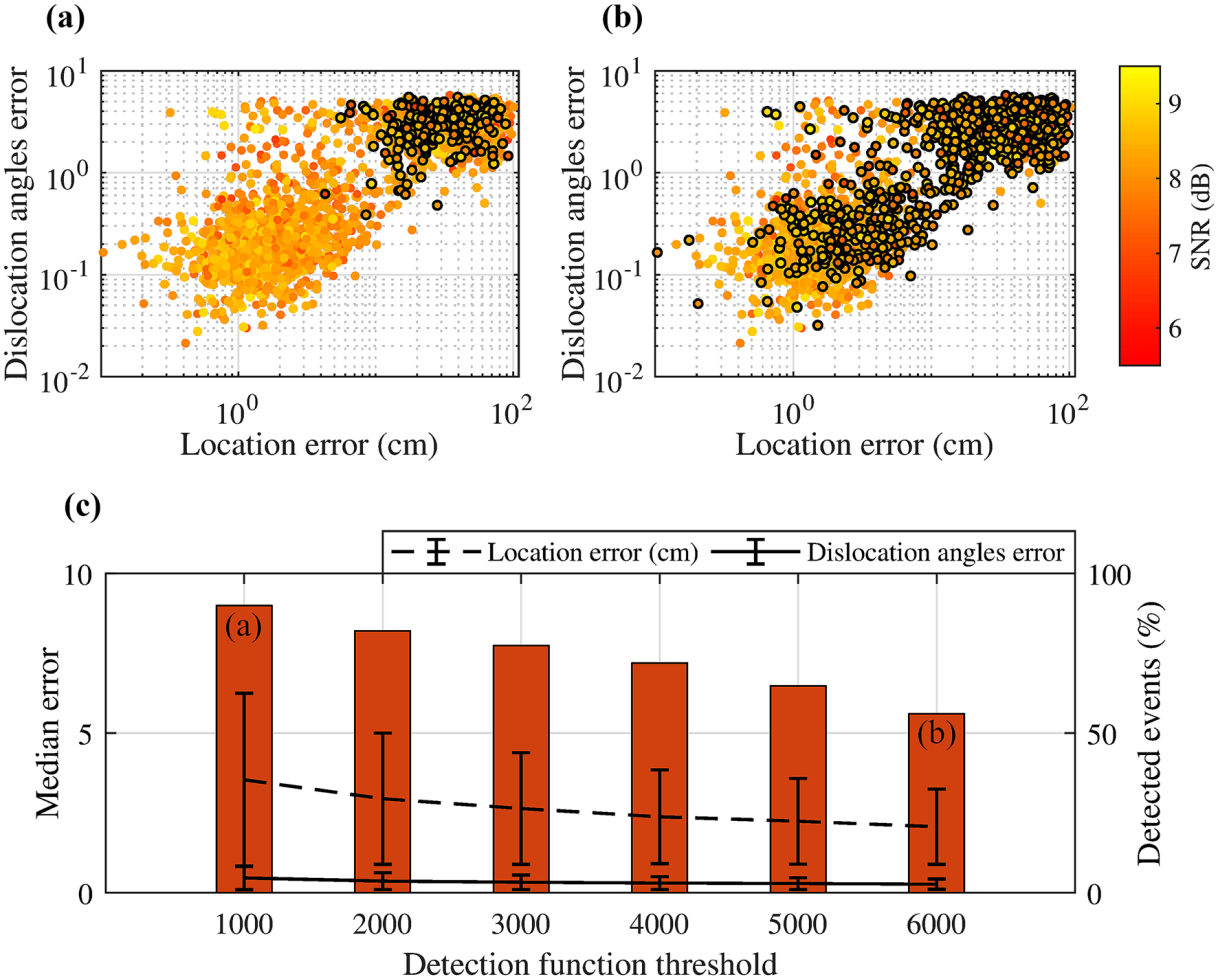
Evaluation of deepCoSeL model. Location and dislocation angles errors for detection thresholds of (**a**) 1000 and (**b**) 6000. Circles with a black contour are events under the threshold (i.e., not detected). (**c**) Median errors in location and dislocation angles for different thresholds applied to the detection function. The bars represent the percentage of events that were detected in each case.

As it is expected, increasing the detection threshold reduces the number of examples that are detected (Fig. 5). In this case, the examples with larger prediction errors anticorrelate with the peak amplitudes of the detection function; thus, demonstrating its effectiveness. For a detection threshold of 1000 (Fig. 5a and c), 90% of the examples are detected, however, the detections include many examples from a cloud that concentrates the larger errors; thus increasing the total median errors. On the other hand, increasing the detection threshold to 6000 (Fig. 5b and c) reduces the total median errors because many less examples from this cloud are detected but that also decreases detection to only 55% of the examples. Thus, we have a trade off between detectability and accuracy of the estimated source parameters, which is in line with other standard processing methodologies. Median location errors lie on the order of a few centimeters, while median angle errors are generally under 5$^\circ$. These errors reflect not only the SNR, but also the uncertainties in the velocities of wave propagation in the medium that were considered for the modeling of the training examples.

In a final test, a set of 500 examples of band-limited random noise were also processed with the deepCoSeL model. The detection function in this case presented a peak value of 31 with a mean of 17. These low values show a reasonable gap between the detection peaks produced by noise and signal, again reinforcing confidence in the effectiveness of the detection function.

## Results

**Figure 6:**
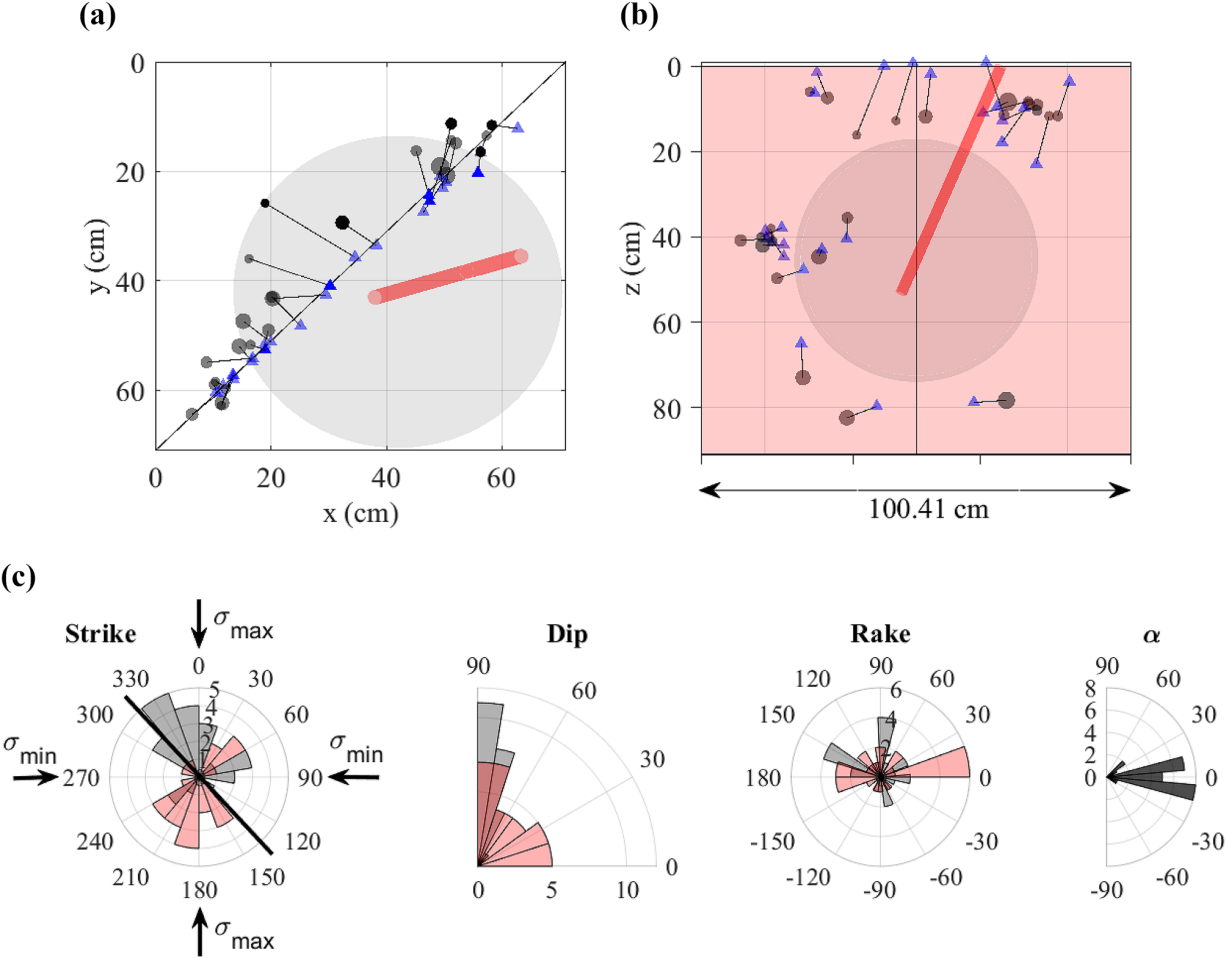
deepCoSeL results for selected real data examples detected in continuous monitoring mode. Views from (**a**) top and (**b**) perpendicular to the artificial cut showing locations from deepCoSeL (circles) and a standard method (triangles). Corresponding events are joined by lines and the size of the circles is relative to the strenght of the detection function. The grey sphere represents the saturated region within the block. (**c**) Fracture angles from the biaxial decomposition of deepCoSeL moment tensor solutions. The orientation of the artificial cut is represented with a thick line over the plot of Strikes. The vertical direction also had applied the same stress as $\sigma _{min}$.

Figure 6a and b present deepCoSeL locations for 25 real data examples that presented detection peaks between $\sim$ 2600 and $\sim$ 5000. These locations fall mostly within 8 cm of the positions estimated with the standard location method. Interestingly, the events with the largest discrepancy in location are those located by the standard method at the upper boundary of the block. As the standard method removes inconsistent time picks iteratively, it is possible that the picks that were finally used to estimate the event location did not provide an adequate constraint in these cases.

The dislocation angles from deepCoSeL can be grouped into two families based on their dip. In this case, one of the families contains mainly semi-vertical fractures approximately aligned with the artificial cut and activating with positive rakes denoting a compression state of stress (see Fig. 6c). This contradicts the stress state at the boundaries of the block at the time of generation of these events. The other family contains fractures with a range of dips mostly aligned with the maximum horizontal stress and activating predominantly in strike-slip mode. The variety of dips responds to the equal minimum stress in the vertical and east-west (i.e., *y*) directions. This family seems more consistent with the state of stresses at the boundaries although it contains more events that did not activate in alignment with the artificial cut.

## Discussion

### Comparison with a standard location method

**Figure 7:**
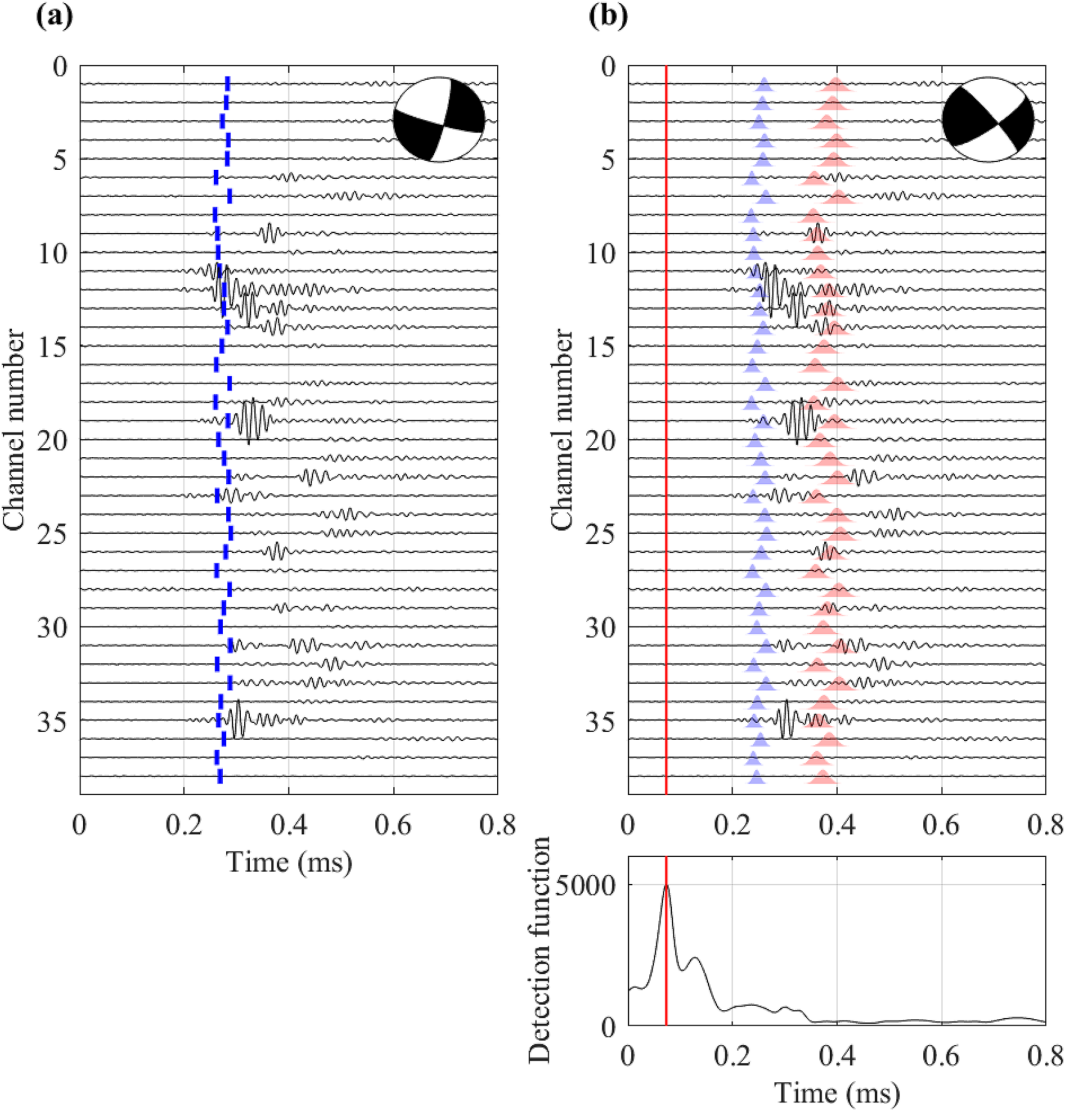
Comparison of theoretical travel times and source mechanism solutions. (**a**) theoretical P travel times for estimated location and fixed velocity model used in the standard method. (**b**) theoretical distributions of P (blue) and S (red) travel times computed based on deepCoSeL location and the distributions of velocities used to train the deepCoSeL model. The red vertical line is the origin time, which corresponds to the peak of the detection function plotted underneath. The difference in location results for this example is 4.2 cm. The beach ball in (**a**) is the fault plane solution derived from a moment tensor estimated with a waveform matching method that does not use data compression. The beach ball in (**b**) is the focal mechanism derived from the deepCoSeL moment tensor solution. Activation in both cases is in strike-slip. In the case of deepCoSeL the solution is aligned with the artificial cut.

The real data used for the testing of deepCoSeL is challenging for several reasons. For instance, the SNR is generally low and influenced by the resonant characteristics of the recording sensors. In addition, many of the trigger examples in the dataset contain the records of more than one source. The standard method attempts to fit a solution to the time picks of the first detected arrivals while deepCoSeL generates detection peaks for all the sets of arrivals that it identifies. In some cases, the arrivals from different sources can be too close to generate distinctive peaks. Fig. 7 presents an example of this scenario, where the first larger peak, which produced a detection, is followed by a smaller peak that did not trigger a detection. Inspection of the waveforms confirms the presence of different arrivals along the same traces, which can correspond to multiple sources.

Figure 7 also highlights some of the differences between deepCoSeL and the standard method. On one hand, the standard method looks only at P-wave information and estimates locations based on a fixed velocity model. If the velocity model is sufficiently accurate, the iterative refinements performed by the standard method can reduce location uncertainty to under 1 cm. On the other hand, deepCoSeL is trained taking into account the uncertainties in the knowledge of the velocity model and environmental noise. This ascribes robustness to deepCoSeL to detect events but also increases the uncertainties in its inferences compared to the standard method.

Another difference between deepCoSeL and the standard method lies in the use of the available information. For instance, if the SNR is low, the standard method cannot use the information because a time pick cannot be defined or the time pick can be deemed inconsistent and discarded. As more time picks are not used in the inversion, the constraint of the location also reduces, thereby increasing uncertainty. This can be a problem in monitoring geometries where sensors happen to be located near nodal planes of the events source mechanism. On the other hand, deepCoSeL is trained to learn the distributions of low and high SNR values for particular combinations of location, source mechanism and monitoring geometry (see for example Fig. 1b). Therefore, all information is used to infer a solution without the need to remove traces and sacrifice constraint. An associated advantage is in the time spent by the standard method to iteratively identify and discard unusable information, which is not required by deepCoSeL.

### Comparison of moment tensor solutions

The average waveform fitting misfit observed with the methodology that does not use compression was 0.59 for the 25 selected AEs. This is a moderately large value that reflects mostly difficulties encountered to associate different P- and S-arrivals to individual events. Although deepCoSeL was not trained with examples that contained multiple events, the behavior of the detection function suggests that it displays some phase association capability in cases with events that present overlapping arrivals (see for example Fig. 7b).

For the solution example presented in Fig. 7 the focal planes derived from the waveform fitting and deepCoSeL solutions present a Kagan angle (i.e., the minimum 3D rotation required to match the two solutions) of 41$^\circ$^86^. Visually, it can be glanced that both solutions represent strike-slips and that the discrepancy is located mostly on the azimuthal orientation. Decomposing the two moment tensor solutions into percentages of isotropic (ISO), compensated linear vector dipole (CLVD) and double couple (DC)^87,88^, both display predominantly DC components with percentages of ISO = 1 %, CLVD = − 15 % and DC = 84 % for the deepCoSeL solution, and ISO = 3 %, CLVD = 12 % and DC = 85 % for the waveform fitting method, again supporting the consistency of both results. For this particular example it can be argued that the deepCoSeL solution is more compelling because it aligns with the artificial cut.

The quality of the results obtained with the waveform fitting method for this particular dataset makes it inadequate as a benchmark to draw more general conclusions on the consistency of the source mechanisms estimated with deepCoSeL. As the ML method also lacks uncertainty metrics, the only reference for evaluation are the results obtained with synthetics during the training and testing of the DCNN. Those results display errors for dislocation angles under $5^\circ$ for high SNR synthetic data examples. Nevertheless, further work with better real data is desirable to investigate in more detail the reliability of source mechanisms derived with deepCoSeL.

### Compression using an autoencoder

An attractive feature of a CS-based compression operator is its generality, which opens the door for its incorporation into the measuring hardware itself. In contrast, alternatives such as principal components (PCA) and autoencoders are adaptive^89,90^; in other words, the compression operator depends on the data itself. Having stated that, recovery of the original data is not satisfactorily achieved with the Green functions dictionary used in CoSeL and it has not yet been attempted as the target output of deepCoSeL. It is also outside the expertise of the authors to comment on the practicality to design a CS-based instrument that can record compressed seismic traces. Current results suggest that deepCoSeL may only be an alternative for fast response and/or fast data scanning to identify periods of time where the uncompressed data is worth analysing in more detail.

As a benchmark for comparison, we tested the source parameter estimation using an autoencoder for data compression. Autoencoders have already been investigated in the past to compress seismic traces^91,92^. In our implementation, the autoencoder consisted of six 2D convolutional layers that performed the encoding followed by six 2D transpose convolutional layers that performed the decoding, and a final 2D convolutional layer that provided the output. All the layers, except for the final one, were part of blocks that included batch normalization, swish activation function and dropout of 0.2. The encoder part of the network had strides and filter sizes designed to compress the data to the same $6.25\%$ used in our deepCoSeL example of application. The training of the autoencoder used two hundred thousand examples per epoch randomly taken from a pool of two million synthetic, noisy examples. During training, the learning rate was reduced when no improvements were observed after three epochs. The training itself stopped when no improvements were observed after five epochs. For practical purposes, the solution space for strike, dip and rake in the training examples was reduced to half the possible ranges for these angles. Using the full solution space required a larger training set, which increases significantly the computational cost to train the autoencoder as it requires the training set in uncompressed size.

The same DCNN used for deepCoSeL was then trained with a training set of two million examples compressed with the encoder part of the autoencoder, again with a reduced solution space for strike, dip and rake for consistency. Testing errors for all the estimated source parameters were slightly larger than for the deepCoSeL model but not by a significant margin. Although the autoencoder was prepared based on reasonable choices, it is likely that its design and hyperparameters could be tuned to match the performance of the deepCoSeL model. Therefore, both approaches could be considered as equivalent alternatives in terms of the results that they provide.

The computational work and time involved in preparing an autoencoder represent its main disadvantage with respect to a CS operator, which in our example required a couple lines of code to create, no training and only two hyperparameters to tune (i.e., input and compressed data sizes). In contrast, the autoencoder requires considerably more computing power, time, expertise, and data for its design and training. Furthermore, the CS operator uses a fraction of the disk space to store and of the time to apply needed by a multilayer autoencoder. These differences could have implications of significance for edge computing implementations. An important advantage of the autoencoder, on the other hand, is the possibility to reconstruct the data, which although it is an integral part of CS theory, it has not been within the scope of the development of deepCoSeL. A line of ongoing research consists in implementing a hybrid approach that uses a CS-encoding operator with a ML-decoder.

### Response time

Other attractive features of deepCoSeL are the fast processing times and the fact that the results include an inference of the source moment tensor. Traditional estimations of moment tensors require analyses that in many cases, and even in more recent ML applications^60,93^, require pre-identification of P and S phases. For example^94^, describes a methodology with similarities to that presented in this work, where a neural network is trained with synthetic examples modeled over a grid of virtual sources. Besides minor differences in the preparation of the training sets, the authors do not consider compression and only the moment tensor is estimated. On the other hand^94^, includes estimations of uncertainty which is an important parameter for the evaluation of results and that is not yet incorporated within deepCoSeL.

The increase in response time introduced by additional analyses to estimate the source mechanism prevents other methodologies from working with a continuous data flow. Instead, they rely on separate routines that feed them with triggered/pre-analysed data. deepCoSeL in our example of application displayed a response time of 7 ms per data frame using a Tesla A30 GPU. Although, far from real-time response in the laboratory setting, sampling rates of 0.5 ms are common in field scale applications, which would place deepCoSeL response in the near real-time for similar input data sizes. Neural network libraries are optimized to perform estimations in batches. For instance, the response time of deepCoSeL in batches of 32 data frames was timed at 10 ms. This could make possible a near real-time implementation with a continuous data flow at field scale.

In addition to fast detection and reporting, an important parameter for risk assessment is the event’s magnitude. This is also an area of improvement for deepCoSeL. We speculate that an estimation of event magnitude could be learned by deepCoSeL via the pattern of SNR at the monitoring network if it is reasonable to assume that the background noise remains relatively constant between the training examples and the observations during implementation. For example^59^, obtained estimates of event magnitude following a standard data pre-processing that preserved the low frequency end of the input data up to the corner frequency for a range of magnitudes of interest. It seems therefore reasonable to test deepCoSeL simply adding the event magnitude to the labels during training. Unlike with the laboratory sensors, this test will become relevant in an application where the instrument response of the sensors is well characterized.

### Uncertainties

Uncertainty estimation is key to evaluate the reliability of parameters estimated through inversion^95,96^. In non-ML methods, Bayesian approaches have been used to estimate uncertainties from moment tensor inversions in field scale applications^97–99^. Alternatively, sampling of the solution space via Monte Carlo or semi-random strategies can also be used to reconstruct uncertainty distributions^97,100^. In ML implementations of source mechanism estimation, uncertainties have been evaluated via Bayesian neural networks^94^, where the strategy to estimate uncertainty distributions relied on the perturbation of input parameters (i.e., event location and velocity model).

Although the training set in deepCoSeL already incorporates perturbations in the velocity model (see Fig. 1b), these perturbations only ascribe robustness to the pattern recognition performed by deepCoSeL and cannot be translated to parameter uncertainties beyond the representation of probabilistic arrival times (see Fig. 7b). With the purpose of estimating uncertainties, an alternative would be to train multiple deepCoSeL models with different, fixed velocity models, perform inferences with each of them and reconstruct uncertainty distributions from the results. This is an area of further development and testing as this change to fix the velocity model during training may, on the other hand, impact the robustness in pattern recognition capabilities of deepCoSeL.

## Conclusions

A new method for fast response seismic processing has been developed, which combines the principles of compressive sensing and deep learning. Although here only exemplified with seismological data, the method can be applied to other fields of science, as the main principle is that the compression process preserves the uniqueness of the patterns that represent the observations of a particular physical model. Thus, a neural network can be trained to make unique connections between these compressed patterns and the parameters of the physical model. This is the same principle behind ML, albeit with a lower computational cost for neural network training facilitated by the compressed training examples. Furthermore, it is possible that the CS-compression operator could take the role that autoencoders play in extracting features from input data before performing regression or classification tasks in ML. Although with a much lower implementation cost.

The method is also an example of two mutually enabling technologies: while on one hand deep learning accelerates the decoding of compressed data into information of interest, on the other, compressive sensing reduces the size of training examples, thus facilitating the expansion of the solution spaces that a neural network can learn with the same computational effort.

The method is aimed at the generation of solutions useful for fast decision-making in the monitoring of induced seismicity and seismic hazards, and its inferences will improve with a better knowledge of the medium of propagation and environmental noise conditions. On the other hand, the method suffers the same limitations of standard methodologies in media with complex Green functions, which may require working in the low frequency range to minimize waveform complexity and facilitate more accurate modeling of synthetic training sets. These limitations could also be alleviated with the use of real data examples for training, although it may be difficult to collect real data examples for training that expand solution spaces that are satisfactorily large; perhaps even more difficult would be the generation of accurate labels. The method is also subject to the same observances of any ML-based predictor, for instance, a lack of generalization in neural network training can result in incorrect results or missed observations.

Current areas of improvement and further development of the methodology include the incorporation of the event’s magnitude to the labels of inferred parameters and the estimation of uncertainties. In the first case, it seems reasonable to test the method in its current form and simply add the event magnitude to the inferred parameters before attempting further methodological modifications. In the second case, a straightforward strategy to generate uncertainties would encompass the training of multiple deepCoSeL models for different velocity model candidates which could be then used to generate ensembles of inferences useful to reconstruct uncertainty distributions.

Despite the existing limitations and areas of further development, deepCoSeL displays important advantages over currently available methods. For instance, it provides a practically continuous sampling of the solution space for location and moment tensor which is virtually impossible to achieve for traditional grid-based methods. Similarly, by transferring computational burden to the training stage of the DCNN, the response time is significantly improved compared to iterative solvers. Furthermore, deepCoSeL offers an alternative to reduce response time that encompasses not only the data processing but also the data transmission, something traditionally handled as separated problems.

## Methods

The modeling of training, validation and testing examples, and preparation of the deepCoSeL system for its implementation with the real data example followed these steps:

### Event locations

Location coordinates for synthetic events were drawn at random following uniform distributions and leaving empty spaces of 5 cm from the block boundaries to approximate far-field conditions. For the construction of labels, the coordinates of the center of the block were removed from each set of coordinates and the result was scaled by a value of 38 cm. This produced adimensional parameters distributed within the approximate interval from $-\,1$ to 1.

### Source mechanisms

The solution space for the source mechanism was restricted to the general dislocation model^73^. For the angles of strike, dip and rake, we sampled angles from uniform distributions covering the full solution spaces of $[0,360]^\circ$, $[0,90]^\circ$ and $[-180,180]^\circ$, respectively. In the case of the angle $\alpha$ (describing the deviation of the displacement vector from the dislocation’s plane) we considered only sources close to pure double couples (i.e., $\alpha \sim 0$); thus, $\alpha$ angles were sampled from a normal distribution with mean of zero and standard deviation of 10$^\circ$.

### Source time function

The source time function was extracted from a high signal-to-noise (SNR) ratio AE recorded during the experiment. For this purpose, the seismogram was low-pass filtered using a third-order Butterworth filter with 80 kHz cut-off frequency. At this frequency cut-off the longer period shear arrivals homogenised their frequency content with the compressional arrivals, such that the same wavelet could be used to model both arrival types. Reducing the frequency content for the processing also had the purpose of reducing the size of the training set required to train the DCNN, which can significantly increase if the complete useful frequency band of about 160 kHz would have been considered^71^.

### Velocity model and seismogram modeling

Synthetic seismograms were modeled via analytical solutions in homogeneous, isotropic media; although, for each source, the medium velocities were sampled from Gaussian distributions with means of $v_P = 2738$ m/s and $v_S = 1580$ m/s for compressional and shear waves, respectively. In both cases, the standard deviation was 4% of the mean. Sampling velocities in this way is meant to capture uncertainties in their variation that results from heterogeneities and stress-induced anisotropy in the rock^84^. While drawing propagation velocities from the probability distributions, it was ensured that the $v_P/v_S$ ratio ranged within the interval (1.45, 2.0), which was empirically selected as reasonable. Synthetics were modeled with a sampling rate of 0.4 $\upmu$s and cut to durations of 2048 samples following real data parameters. The density of the block was fixed at $\rho = 2000$ $\mathrm{kg/m}^3$.

### Signal-to-noise ratio modeling

Noise was modeled using Gaussian time series with mean of zero and filtered with the same low-pass as the synthetics. The standard deviation in the time series was set per channel using mean values extracted from the root-mean-square (RMS) amplitudes estimated within 128-sample windows in all the available AE trigger files. This part of the modeling helped to approximate the background noise level at individual receivers.

Afterwards, sets of 500 synthetics were modeled with varying amplitude-scaling factors and added to the noise time series to approximate the ranges of values observed in the histograms of peak amplitude and SNR in the AE triggers (SNR is defined here as the ratio between the peak amplitude over the RMS of a complete trace or trigger). The locations and source mechanisms for this modeling were generated following the same procedures described in previous sections. The histograms of peak amplitude and SNR in the observations were reasonably approximated using scaling factors drawn from a uniform distribution in the interval [2e14, 2.3e15]. These scaling factors are related to the seismic moment (i.e., $M_0$) of the AEs, however, they cannot be referred to as $M_0$ because the instrument response of the sensors was not available to calibrate the observations. The drawing of the scaling factors considered a uniform distribution rather than a Gutenberg-Richter distribution because the objective was to present the DCNN with an even number of low and high magnitude examples for its training.

### Compression and normalization

The compression operator ${\varvec{\Phi }}$ was prepared by drawing iid samples from a Gaussian distribution with zero mean and standard deviation of 1/2048. Its dimensions were $128\times 2048$, which represents a data compression down to 6.25%. The compression level was chosen based on previous synthetic modeling results, which reported degradation in deepCoSeL predictions for compression below $\sim 3\%$^71^. After compressing a simulated data example with ${\varvec{\Phi }}$, its sample amplitudes were scaled to a dynamic range of 0–255, and saved as a 16-bit integer portable network graphics image (i.e., png extension). Databases for training, validation and testing were created following these steps, each containing five million, ten thousand and ten thousand images, respectively. As with the compression level, the number of examples in the training set was selected based on previous modeling results with synthetics^71^.

### DCNN architecture, training and predictions

**Figure 8:**
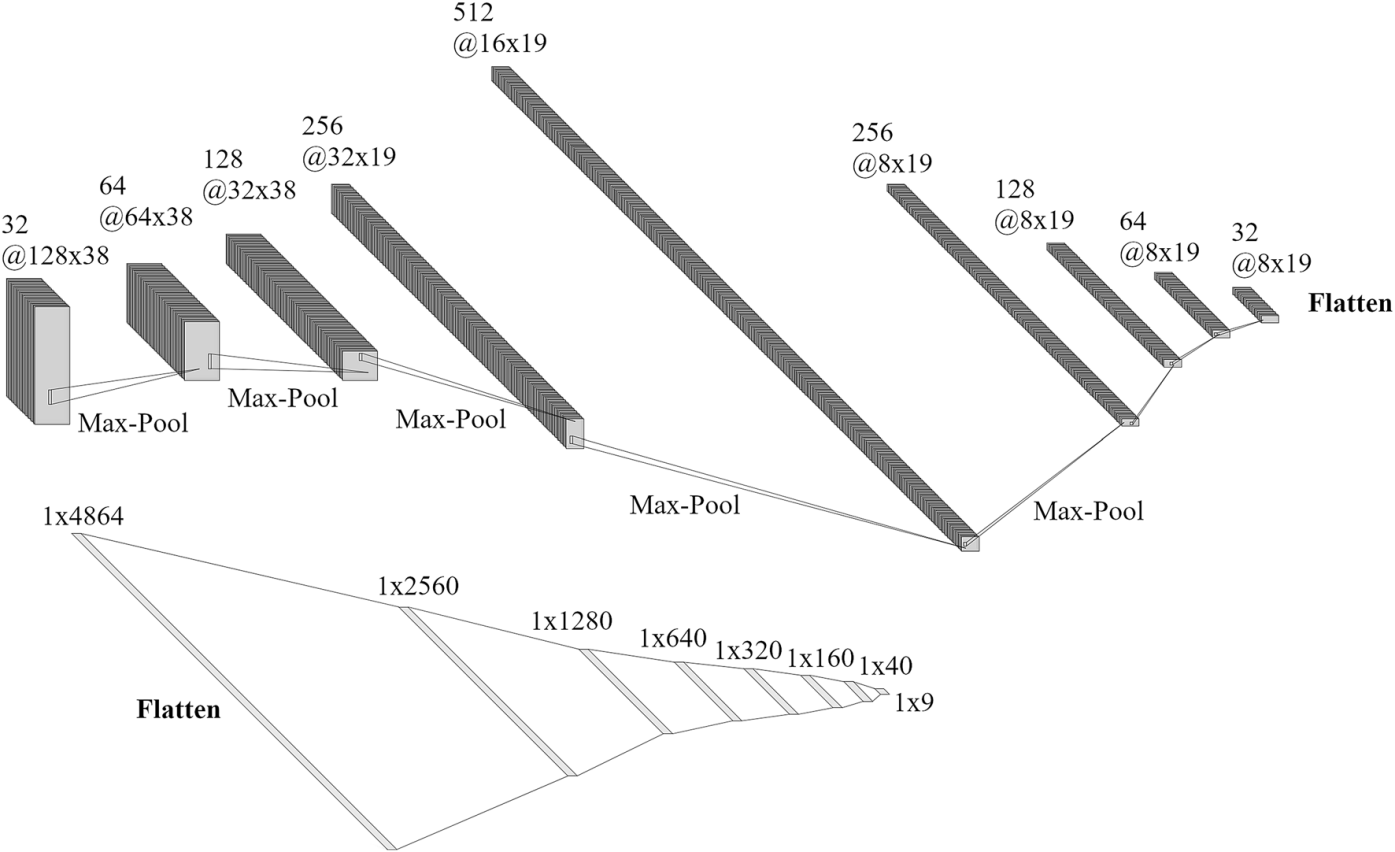
DCNN architecture used in this work. Top: first part of the network with convolutional and pooling layers. After the last convolutional layer the output is flattened and input into a fully connected network (bottom). All layer outputs, except for the output layer, are batch-normalized and activated with a swish function. This figure was prepared using schematics drawed with NN-SVG^106^.

The design and training of the DCNN used the Keras application programming interface as contained in the Tensorflow open source platform^101,102^. The DCNN architecture was defined based on user-experience and trial-and-error. It consisted of a series of convolutional and pooling layers followed by fully connected layers (Fig. 8). Batch normalization^103^ was applied to the output of every convolutional and dense layer before the application of a swish activation function^104^. Only the output layer did not have these two operations applied. The batch size for training was 512 images. Learning performance was measured with a mean square error and the optimization was executed using the Adamax algorithm^105^ with a learning rate of 0.01 during the first 80 epochs and 0.001 during the last 20. During learning, we also halted the training every 5 epochs to evaluate the model over testing sets of 10000 examples. From these evaluations we tracked the improvements in location and dislocation angles errors as additional metrics to evaluate when the DCNN stopped learning. The model took about two days to train using a NVIDIA A30 GPU unit.

The DCNN in this application takes a set of compressed seismograms of dimensions $128 \times 38$ and treats them as a one-channel image (see Fig. 8). Before entering the DCNN, training image amplitudes were scaled to the range $0-1$ and their mean was removed. For prediction purposes, the seismograms were preprocessed following the same steps as during the preparation of training examples. This implied two scaling steps, the first one (scaling to $0-255$ range) followed by the quantization of amplitudes to 16-bit integers, which were then returned to floating point numbers by the second scaling operation (scaling to $0-1$ range). These redundant preprocessing steps were retained for consistency and in order to fit the application to a standard Tensorflow workflow. The output from the DCNN were nine parameters. The three parameters that correspond to the source location were transformed back from the adimensional label space to the spatial coordinate system of the medium. The six parameters that correspond to the source moment tensor were transformed to dislocation angles using the biaxial decomposition^74^.

## Data Availability

The datasets generated during and/or analysed during the current study are available from the corresponding author on reasonable request.
